# Atomic force microscopy measurements of lens elasticity in monkey eyes

**Published:** 2007-04-02

**Authors:** Noël M. Ziebarth, Ewa P. Wojcikiewicz, Fabrice Manns, Vincent T. Moy, Jean-Marie Parel

**Affiliations:** 1 The Ophthalmic Biophysics Center, Bascom Palmer Eye Institute, University of Miami Miller School of Medicine, Miami, FL; 2 Biomedical Optics and Laser Laboratory, Department of Biomedical Engineering, University of Miami College of Engineering, Coral Gables, FL; 3 Department of Physiology and Biophysics, University of Miami Miller School of Medicine, Miami, FL; 4 University of Liege Department of Ophthalmology, CHU Sart-Tillman, Liege, Belgium; 5 Vision CRC, University of New South Wales, Sydney, Australia

## Abstract

**Purpose:**

To demonstrate the feasibility of measuring the elasticity of intact crystalline lenses using atomic force microscopy (AFM).

**Methods:**

AFM elasticity measurements were performed on intact lenses from 18 fresh cynomolgus monkey cadaver eyes (4-10 years old, <1 day postmortem) that had been left attached to their zonule-ciliary body-sclera framework. The eyes were prepared by bonding a plastic ring on the sclera after removal of the conjunctival, adipose, and muscle tissues. The posterior pole was sectioned, with the excess vitreous removed, and the eye's anterior section was placed on a Teflon slide to protect the posterior pole of the lens. The cornea and iris were then sectioned. The lens-zonule-ciliary body-sclera section was then placed in a Petri dish filled with balanced salt solution in an AFM system designed for force measurements. Next, the central pole of the anterior surface of the intact lens was probed with the AFM cantilever tip. The recorded AFM cantilever deflection-indentation curves were used to derive force-indentation curves for the lens after factoring out the deflection of the cantilever on a hard surface. Young's modulus of the lens was calculated from the force-indentation relation using the Hertz model.

**Results:**

Young's modulus was 1,720±880 Pa (range: 409-3,210 Pa) in the 18 cynomolgus monkey lenses.

**Conclusions:**

AFM can be used to provide measurements of the elasticity of the whole lens including the capsule. Values obtained using AFM on cynomolgus monkey lenses are similar to published values obtained using dynamic mechanical analysis on young human lenses.

## Introduction

Presbyopia is the progressive loss of accommodation with age [1-4]. Even though the exact causes of presbyopia are still not fully understood, it is generally believed that its origins are multifactorial and involve several of the accommodative structures, including the lens, ciliary muscle, ciliary body, and zonules. A number of studies suggest that presbyopia entails a loss of lens elasticity with age [5-8].

The elasticity of the lens has been previously investigated using a spinning method [5], uniaxial stretching [9], squeezing [6], and dynamic mechanical analysis [7,8,10]. The spinning method, uniaxial stretching, and squeezing provided relative values of lens elasticity that could be used to study age-related changes but did not provide absolute values needed for mechanical models. Dynamic mechanical analysis (DMA) provides absolute values of lens elasticity but it requires special attention during the calibration of the device and the preparation of the tissue. Heys et al. [7] performed measurements on human lenses that were frozen at -80 °C, partially thawed, sectioned equatorially, and then cored using an 8.5 mm internal diameter trephine. Measurements were performed at 22 °C, and dehydration was prevented using moistened foam rubber surrounding the probe. Weeber et al. [8] performed measurements on human lenses that were frozen at -70 °C, defrosted, and then sectioned. Measurements were performed at 36 °C, and dehydration was prevented using silicone oil. Published values of lens elasticity measured using DMA differed by several orders of magnitude in older lenses, most likely due to these differences in methodology [7,8].

Another technique that could provide insight into lens elasticity and changes with age is atomic force microscopy (AFM). In medicine and biology, AFM has been used previously for elasticity measurements of individual cells [11-16], proteins [17,18], and soft tissue [19]. The purpose of the present study was to demonstrate the feasibility of using AFM to measure local in situ lens elasticity in a manner that is atraumatic to the tissue.

## Methods

### Atomic force microscope

**Figure 1:**
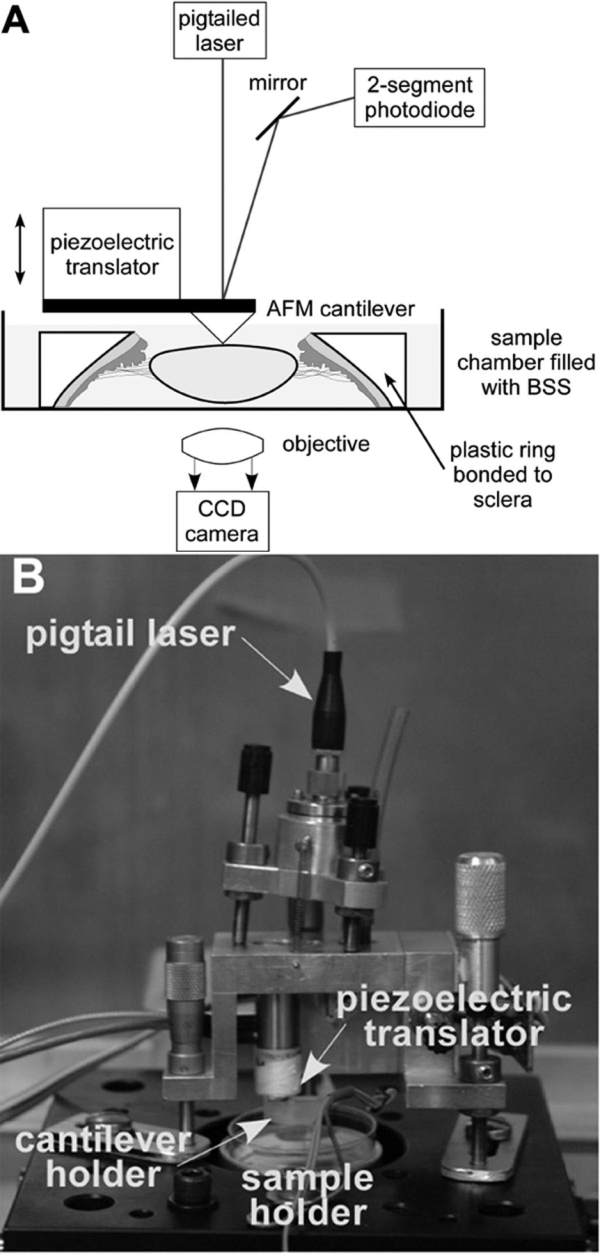
Atomic force microscope for elasticity measurements. The atomic force microscopy (AFM) system for elasticity measurements is a laboratory-made modification of the AFM design used for imaging [15,20]. It is shielded inside an acoustic/vibration isolation chamber. **A**: The cantilever is moved vertically using a piezoelectric translator that responds to applied voltage. A Petri dish containing the lens is placed below the cantilever, and the cantilever is lowered onto the lens. The cantilever is bent, causing the beam of the laser diode to be deflected. A photodiode monitors these deflections. Custom software controls the piezoelectric translator and times the measurements. **B**: Shown is a labeled photograph of the AFM used for lens capsule elasticity measurements.

The AFM system used was a laboratory-made modification of the AFM design used for imaging [20,21] (Figure 1). It was shielded inside an acoustic/vibration isolation chamber. The AFM cantilever tip (60 nm gold coating, 0.2 mm tip, MLCT-AUHW, Veeco, Santa Barbara, CA) was lowered onto the sample at a rate of 5 mm/s. A piezoelectric mechanism (Physik Instrumente, Karlsruhe/Palmbach, Germany) moved the cantilever vertically in response to applied voltage. During the elasticity measurements, the cantilever was lowered onto the sample and underwent bending following contact with the sample. The degree of this bending was related to the mechanical properties of the sample: the harder the sample, the more the cantilever bent. The beam of a diode laser was reflected off the cantilever surface and underwent deflection in response to the cantilever bending. The cantilever deflections were monitored by a position-sensitive two segment photodiode (UDT Sensors, Hawthorne, CA). Custom software controlled the piezoelectric translator and timing of the measurements.

### Calibration of the atomic force microscope cantilever

**Figure 2:**
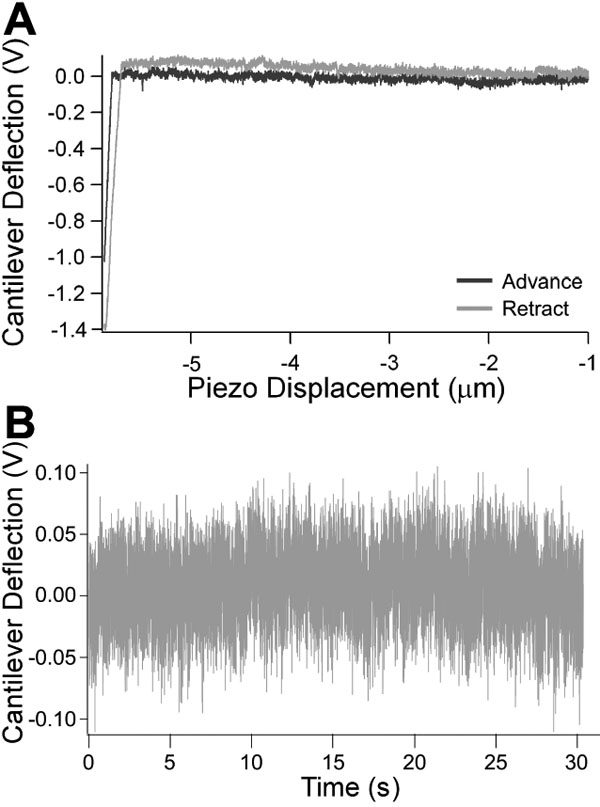
Calibration scans performed to characterize the atomic force microscope cantilever. **A**: Force scan conducted on a hard surface to determine voltage-deflection relationship. A force scan was conducted on the surface of the Petri dish with no sample to determine the relationship between voltage detected at the photodiode and cantilever deflection. The curve provides the voltage-displacement curve in the absence of indentation. This response is used in the calculation of the force-indentation curves obtained on a samples. **B**: Recording of natural vibrational frequency of cantilever in fluid. The thermally induced cantilever fluctuations are measured by recording the vibrational frequency of the cantilever in fluid. This response is used to measure the spring constant using equation 1.

Each AFM cantilever was calibrated before an experiment to determine its spring constant [22]. This was accomplished by first recording the voltage detected at the photodiode due to deflection of the cantilever as a function of piezoelectric displacement to determine the relationship between voltage and cantilever deflection. This scan was conducted by placing the cantilever in contact with the bottom of a Petri dish with BSS with an indentation force of 1 nN (Figure 2A). To determine the thermally induced fluctuation of the cantilever, we lowered the cantilever tip so that it was submerged in BSS, but not touching the bottom of the Petri dish (Figure 2B). Equation 1 shows the measured variance of the deflection used to calculate the spring constant:

$$k_{C}=\frac{k_{B}T}{<\chi^{2}>}$$

where *k_B_* is Boltzmann's constant, *T* is temperature, and <*Χ^2^*> is the variance of the cantilever deflection. The spring constants obtained using this methodology were consistent with the nominal value of 10 mN/m given by the manufacturer (Veeco).

### Experimental protocol

AFM measurements were made on the central anterior surface of 18 intact lenses (six pairs, six unpaired) from healthy cynomolgus monkeys (*Macaca Fascicularis*, 7.2±2.1 years, range: 4.2-10 years). Enucleated monkey eyes were obtained from the University of Miami Division of Veterinary Resources following approved institutional animal care guidelines through an approved tissue-sharing protocol. Eyes were obtained from monkeys euthanized for experiments not related to the current study. After enucleation, all eyes were placed in sealed containers with gauze soaked with BSS to prevent dehydration of the globe. All eyes were stored at 5 °C and returned to room temperature before they were used. Experiments were performed no more than one day postmortem (0.25±0.27days).

**Figure 3:**
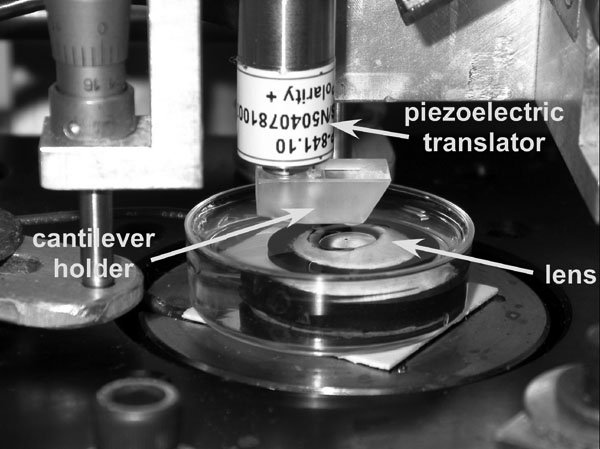
Location of the lens sample in the atomic force microscopy system. Location of the lens sample in the atomic force microscopy system. The lens sample is placed in a Petri dish filled with DMEM. The dish with the lens is then placed in the AFM system under a PMMA block that contains the AFM cantilever.

**Figure 4:**
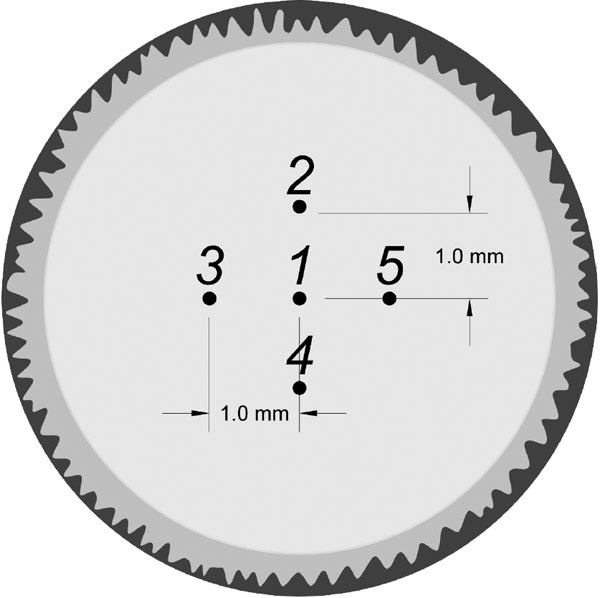
Measurement locations used to validate the visual lens positioning technique. Measurement locations used to validate the visual lens positioning technique. The same lens was measured in five different locations around the lens center. These measurements were used to validate the sample positioning technique and to determine the effect of probe positioning on the variability of the measurements.

The protocol described as follows was used to prepare the lens for AFM measurements. A custom-made circular black plastic ring was machined to fit the average globe radius of curvature (9.5 mm). The ring was bonded onto the sclera in the region of the ciliary body approximately 2 mm posterior to the limbus using cyanoacrylate adhesive (Duro Quick Gel super glue, Loctite Corp., Rocky Hill, CT) after the conjunctival, adipose, and muscle tissues were removed. The ring enabled dissection of the globe with minimal deformation while keeping the ciliary body-zonule-lens framework intact. Dissection was not initiated until the glue had dried. This was to ensure that the glue fumes did not cause any surface dehydration of the lens. The posterior pole was removed by making a circumferential incision through the sclera. Excess vitreous was carefully removed, and the eye section was placed on a Teflon slide. The cornea and iris were then sectioned. The clinical appearance of each lens was examined under the operation microscope. All lenses were noted to be intact and clear. The posterior pole of the lens remained intact in all eyes. The mounted tissue specimen was then placed in a Petri dish filled with BSS under the polymethylmethacrylate block containing the AFM cantilever (Figure 3). The lens was positioned visually so that the cantilever tip was over the central pole of the anterior surface. The tip was lowered until it just touched the surface of the lens. This position was determined by the point when the reflected laser beam moves. The tip was then lowered, using the piezoelectric control, so that it was in a position to probe the surface of the lens. The measurements were conducted using an indentation force of 300 pN, 0.25 s of contact time with the sample, and a cantilever retraction speed of 5 mm/s. The voltage detected at the photodiode due to deflection of the cantilever was recorded as a function of piezoelectric displacement. These recordings were repeated approximately 10 times per lens. The lens positioning technique was validated by measuring one cynomolgus monkey intact lens in five different locations around the center (Figure 4). The same protocol was used to demonstrate that the AFM could provide reliable measurements on a sample with a shape similar to that of the lens and mounted with the same approach. This was done by measuring a silicone intraocular lens of known modulus of elasticity.

### Data analysis

**Figure 5:**
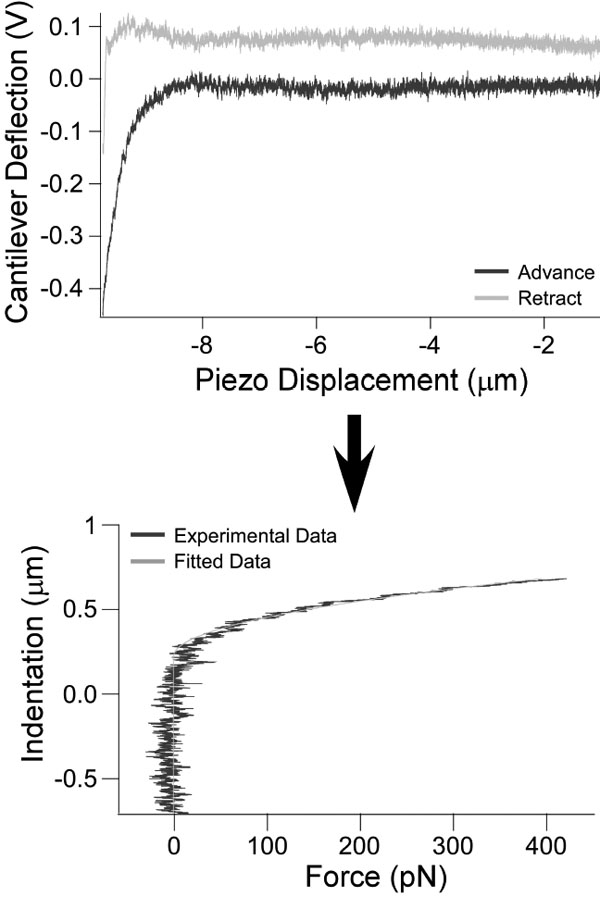
Analysis process for atomic force microscopy measurements. Force scans are taken by probing the sample with the cantilever and recording the cantilever deflection (upper panel). Force (in picoNewtons) versus indentation was derived from the cantilever spring constant and slope found during calibration (lower panel). Young's modulus was then calculated using the Hertz model.

The lens indentation was calculated by subtracting the piezo displacement when probing the sample from the piezo displacement when probing the hard Petri dish. Force was calculated from the spring constant (k_C_, mN/m) and slope of the cantilever deflection versus piezo displacement relationship (C, m/V) found during calibration using the following equation:

$$F=k_{C}C\Delta V$$

where Δ*V* is the change in voltage (*V*) recorded during the scans (Figure 5). The elastic modulus was found using the Hertz Model [23]:

$$F=\frac{K}{2\left(1-v^{2}\right)}\frac{4}{\pi \tan\theta }\alpha^{2}$$

where *F* is the measured force (*N*), *K* is Young's modulus (Pa), *v* is Poisson's ratio (*v*=0.5), θ is the angle of indentation (normally assumed to be 55°), and α is the measured indentation (m). The determination of Young's modulus was carried out by least square analysis of the measured force-indentation curves using data analysis software (OriginLab Corporation, Northampton, MA). Each curve fit was verified visually. The average of the values was then used as the modulus for that sample.

## Results

All values of Young's modulus recorded (Table 1) at the different positions around the center of the lens were between 6% and 35% of the values recorded at Position 1.

**Table 1:** Young's modulus obtained at five different locations around the center of an intact cynomolgus monkey lens.

	**Position 1**	**Position 2**	**Position 3**	**Position 4**	**Position 5**
Average±SD (Pa)	1259±97	1430±226	1568±315	1680±499	1338±301
Range (Pa)	1115-1381	1221-1962	1042-1942	1174-2477	1042-1922
% Difference	-	13.6	24.5	33.4	6.3

Young's modulus obtained at five different locations around the center of an intact cynomolgus monkey lens. Young's modulus was measured in the center and at four additional locations 1mm from the center for one cynomolgus monkey lens (see Figure 4). The values at the different locations were within 35% of the original measurement at the center.

AFM measurements were performed on a silicone intraocular lens with a Young's modulus of 3.55 MPa (determined by dynamic mechanical analysis). Our AFM system showed the modulus was 3.87 MPa. This is a percent difference of 9%.

Young's modulus was 1,720±882 Pa (range: 409-3,210 Pa) in the 18 cynomolgus lenses (Table 2).

**Table 2:** Young's modulus of elasticity obtained for 18 cynomolgus monkeys.

**Lens**	**Age (years)**	**Postmortem Time (hours)**	**Young's Modulus (average±SD, Pa)**	**Variability (%)**
1 OD	4.2	24	409+108	26.4
2 OD	4.5	24	939+150	16
2 OS	4.5	3	3210±370	11.5
3 OD	5.2	5	917±41	4.5
4 OD	5.4	3	746±303	40.5
4 OS	5.4	5	S60±125	14.5
5 OD	6.5	5	2479+194	7.8
5 OS	6.5	3	2678+216	8.1
6 OD	6.7	3	2305+616	26.7
6 OS	6.7	3	1710±417	24.4
7 OD	7.8	3	2061±426	20.7
7 OS	7.8	3	2862+347	12.1
8 OD	9.2	5	1259±97	7.7
9 OD	9.4	5	1051±90	8.6
10 OD	9.7	5	2440±360	14.8
11 OS	10	24	1656±359	21.7
12 OD	10	3	726±144	19.8
12 OS	10	5	2647±859	32.5
		AVERAGE	1720±S80Pa	17.7±9.7%

Each lens measurement was repeated 10 times successively. The variability was defined as standard deviation divided by the average multiplied by 100%.

## Discussion

This study demonstrated the feasibility of using AFM for whole lens elasticity measurements in situ. For 18 cynomolgus eyes, we found an elastic modulus between 409 and 3,210 Pa. Since the AFM tip indents the lens capsule, we initially thought that the values obtained would correspond to the elasticity of the lens capsule alone. Previous studies [24-26] showed the lens capsule had an elastic modulus ranging from 0.3 to 6 MPa. These values were three orders of magnitude greater than the values found in the current study. This large difference suggested that the AFM measured the Young's modulus of the entire anterior portion of the lens, including the capsule, epithelial cells, and cortex. This indicated that the capsule became deformed in bending mode under the pressure of the AFM tip, which required much less force than deforming the lens matter in compression mode. If this hypothesis is correct, then the force exerted on the sample by the AFM tip indented only the softest part of the lens: the cortex. However, it is likely that the presence of the lens capsule influenced the results.

The results obtained on the intraocular lens confirmed that the AFM provided accurate values of elasticity on samples with a shape similar to that of the lens and that the modulus of elasticity corresponded to the bulk modulus of elasticity of the sample. There are currently no intraocular lenses or commercial lenses available with an elastic modulus similar to that of the human crystalline lens. The modulus of elasticity of the intraocular lens is approximately three orders of magnitude greater than that of the crystalline lens. The conclusions from the experiments on the intraocular lens can therefore not necessarily be applied to the crystalline lens. Additional studies on more suitable models of the lens are needed to confirm these findings.

For this feasibility study, AFM elasticity measurements were performed on cadaver cynomolgus monkey lenses. Monkey lenses were used because they were readily available to our laboratory through tissue-sharing protocols from other research studies. The monkey eyes were received immediately after euthanasia, which ensured that measurements were obtained on fresh tissue. This eliminated potential measurement artifacts due to lens swelling and water uptake that can occur in human eyes obtained from eye banks because of storage conditions and increased postmortem time [27]. Leaving the lens in the eye does permit ion and water entry into the lens. However, our protocol was based on previous studies on lens preservation [27], which showed that there is no water uptake in fresh monkey lenses. The immersion in DMEM will prevent or reverse this effect if the postmortem time is short, as it was in this study.

To the best of our knowledge, the only data available on the elastic properties of the lens have been obtained on human tissue. Monkeys have a lens structure, composition, and accommodative mechanism that is qualitatively similar to that of the human [28-35]. The lens elasticity in prepresbyopic monkeys and humans should therefore be similar. Heys et al. [7] provided values of the shear modulus of elasticity (G) and Weeber et al. [8] provided values for the compliance (1/G) for the cortex of human lenses. For comparison, these values were converted to Young's modulus (*E*) using the following relationship: *E*=2*G*(1+ν), where ν is the Poisson ratio (assumed to be 0.5). Our values were comparable to those obtained by Heys et al. [7] and Weeber et al. [8] for the cortex of prepresbyopic human lenses (98.3±64.5 Pa less than 30 years old [7]; 530-5,300 Pa less than 40 years old [8]; Table 3). These findings suggest that AFM provides values similar to those obtained using DMA despite differences in the approach and potential differences between human and monkey lens elasticity. The values obtained with AFM on monkey eyes are within the range that would be expected under the assumption that human and monkey lenses have similar properties.

**Table 3:** Summary of lens elasticity measurements.

**Researcher**	**Method**	**Species (Age range)**	**Samples**	**Young's Modulus (Pa)**
Fisher, 1971 [5]	Lens spinning	Human (0-67 years)	40	432-3634
Heys, 2004 [7]	DMA	Human (14-76 years)	18	145.5-7731
Weeber, 2005 [8]	DMA	Human (18-90 years)	39	533-950,000
Current study	AFM	Cynomolgus (4-10 years)	14	409-3210

The values provided for Fisher correspond to the polar elasticity of the whole lens, whereas the values provided for Heys et al. [7] and Weeber et al. [8] are for the lens cortex only.

Ten successive measurements were performed on each lens and then averaged to provide Young's modulus for that lens. The variability of repeated measurements of the same lens (standard deviation divided by average) ranged from 4.5% to 40.5%, with an average of 17.7% for cynomolgus monkey eyes. This variability is within the same range of the variability of the DMA results. Heys et al. [7] reported a variability of up to 30%, and Weeber et al. [8] reported a variability average of 6%. A number of factors could affect the variability of the measurements, including changes in hydration as well as inherent changes in the tissue postmortem.

From the experiment measuring Young's modulus of the same lens in different locations around the center, we found differences up to 35% due solely to change in position. This effect of position on Young's modulus most likely contributed to the differences we encountered with paired eyes as well as between animal variability. However, this 35% variance does not account for the large range of values obtained. The measured elastic modulus varies by approximately eight times between the smallest and largest value. The origin of this large variation remains to be investigated. In separate experiments, we found that a large variability in the biometric and optical parameters of cynomolgus monkey eyes obtained from the same source. This variability is also consistent with the results of Weeber et al. [8] on human lenses. They found that the mechanical properties of eyes from different donors of the same age deviated by at least one order of magnitude. This could indicate that the lens mechanical properties have a relatively high variability between donors of the same age. However, it is more likely that the variations are introduced by the tissue preparation, storage, and handling, independent of the measurement technique.

In summary, our results demonstrate the feasibility of using AFM to measure the elasticity of the whole lens including the capsule with minimal disruption of the tissue. The values obtained on cynomolgus monkey lenses are comparable to those obtained using dynamic mechanical analysis on human lenses. Additional studies are under way to quantify the respective contribution of the lens capsule, cortex, and nucleus to the measured modulus of elasticity and quantify the effect of age.
